# Reproducibility and Prognostic Value of WHO1973 and WHO2004 Grading Systems in TaT1 Urothelial Carcinoma of the Urinary Bladder

**DOI:** 10.1371/journal.pone.0083192

**Published:** 2014-01-07

**Authors:** Ok Målfrid Mangrud, Rune Waalen, Einar Gudlaugsson, Ingvild Dalen, Ilker Tasdemir, Emiel A. M. Janssen, Jan P. A. Baak

**Affiliations:** 1 Department of Pathology, Stavanger University Hospital, Stavanger, Norway; 2 Clinical Institute-1, University of Bergen, Bergen, Norway; 3 Department of Pathology, Innlandet Hospital Trust, Lillehammer, Norway; 4 Department of Research, Stavanger University Hospital, Stavanger, Norway; 5 Department of Urology, Stavanger University Hospital, Stavanger, Norway; University of Campinas, Brazil

## Abstract

**Background:**

European treatment guidelines of TaT1 urinary bladder urothelial carcinomas depend highly on stage and WHO1973-grade but grading reproducibility is wanting. The newer WHO2004 grading system is still debated and both systems are currently used.

**Aims:**

To compare reproducibility and prognostic value (of stage progression) of the WHO1973 and WHO2004.

**Methods:**

One hundred and ninety-three primary urothelial carcinomas were reviewed. Follow-up data were retrieved from the patient records. Kappa statistics and Harrell's C-index were used.

**Results:**

Median follow-up was 75 months (range 1–127). 17 patients (9%) progressed, 82% of these within and 18% after 60 months. The distribution of WHO73-grades 1, 2 and 3 was 23%, 51% and 26%, interobserver agreement for each individual grade was 66% (kappa = 0.68), while for grades 1&2 versus 3 89% (kappa = 0.68). Intraobserver reproducibility was 68–63% for WHO73 and 88–89% for WHO73 as 1&2 vs.3. Progression free survival rates at 5 years were 95% (grade 1), 98% (grade 2) and 82% (grade 3) and 96% and 82% for grades 1&2 versus 3 (Hazard Ratio, HR, 5.4, p = 0.003). Using WHO2004, 62% were low grade and 38% high grade, inter-observer agreement 87% (kappa = 0.70), intraobserver reproducibility 93%, and progression free 5-year survival rates 97% and 85% (HR 6.6, p = 0.004). Positive and negative predictive values for stage progression within 5 years for the WHO73 (1&2 vs. 3) were 18% and 96%, and 15% and 97% for the WHO04. Using Harrell's C-index, none of the grading systems was prognostically superior.

**Conclusion:**

None of the grading systems is prognostically stronger than the others. Most importantly, inter-observer reproducibility and sensitivities for stage progression of both systems are low and need improvement for optimal treatment.

## Introduction

**Figure 1:**
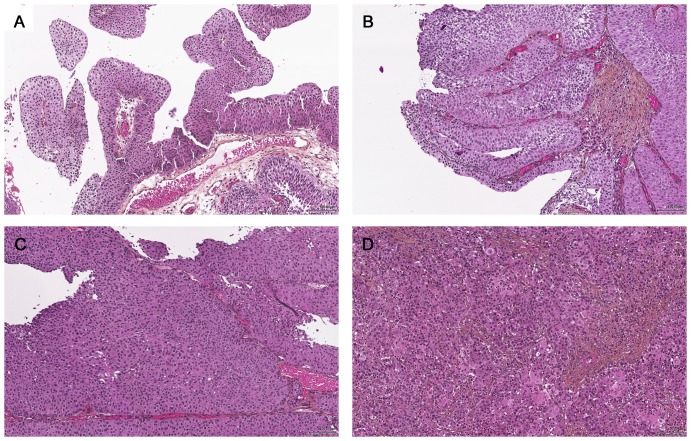
Grading of urothelial carcinomas. **A.** WHO73 Grade 1/WHO04 Low grade. **B.** WHO73 Grade 2/WHO04 Low grade. **C.** WHO73 Grade 2/WHO04 High grade. **D.** WHO73 Grade 3/WHO04 High grade.

Superficial (TaT1) urothelial carcinoma (UC) is the most common urinary bladder cancer in the Western world. Approximately 70% recur and 8–30% progress to a higher T-stage [1], [2]. Prognosis in TaT1 UCs depends largely on lamina propria invasion, and grade. European treatment guidelines [3] are based on the 1973 World Health Organization (WHO73) grading system. The WHO73 discerns three grades (1, 2, and 3) based on the degree of anaplasia [4] (Figure 1) but intra- and inter-observer reproducibility is wanting and efforts have been made to develop a more reliable grading system. Following a WHO/International Society of Urological Pathology (ISUP) consensus conference, a new grading system was introduced in 1998 [5] and adopted in the WHO 2004 blue book (WHO04) [6]. The WHO04 divides the neoplasms into benign papillomas, papillary urothelial neoplasm of low malignant potential (PUNLMP), and low and high grade carcinomas. The WHO04 was thought to be more reproducible than the WHO73, but several studies have shown considerable inter-observer variability [7], [8]. There have also been discussions on the incidence of PUNLMP with rates ranging from 12–39%, and stage progression rates between 2 and 8% [9]–[11], very similar to the low grade carcinomas.

Therefore, the aims of the current study were to compare the inter-observer reproducibility and prognostic value (on stage progression) of the WHO73 and WHO04 in patients with TaT1 urothelial urinary bladder cancer and the clinical significance of distinguishing PUNLMP and low grade cancers.

## Methods

### Ethics statement

The study was approved by the Norwegian Regional Ethics Committee (REK Vest, #106/09) before the start of the study. With approval from REK Vest, informed consent was not obtained as the tissue samples had already been removed for diagnostic and treatment purposes.

### Patients

Two hundred and forty nine consecutive cases of primary (first diagnosis) non-muscle invasive urothelial carcinoma of the urinary bladder were diagnosed at the Departments of Urology and Pathology, Stavanger University Hospital (SUH) January 1, 2002 through December 31, 2006.

Tumour tissue was obtained by transurethral resection or biopsy at the Department of Urology, SUH. All samples were originally routinely diagnosed as primary urothelial carcinoma WHO73 grade 1–3, pTaT1, by seven different pathologists. The tumour tissue was fixed in 4% buffered formaldehyde, dehydrated and embedded in paraffin. Four µm thick sections stained with haematoxylin-erythrosine-saffron were used for routine diagnostics. In total fifty-six cases were excluded from this study; the majority of these due to inadequate sample quality (Table 1).

**Table 1:** Exclusion criteria, number of excluded and included patients.

Primary pTaT1 urothelial carcinomas at SUH 2002–2006	249
Insufficient material	21
Thermal damage	11
Fragmented specimen	1
Necrotic specimen	2
Sarcomatoid differentiation	1
Previous urothelial carcinoma (on review of clinical notes)	1
cT3 or cT4 (on review of clinical notes)	3
pT2 at re-TURV	2
pT2 at review	1
Clinical metastasis at time of diagnosis	2
Lost to follow-up	11
**Included in study**	**193**

The patients were uniformly treated according to the national guidelines at the time of diagnosis. All patients underwent transurethral resection (TUR) followed by a single instillation of a cytotoxic agent (normally 40 mg Mitomycin C). High risk patients were treated with BCG-instillations (alternatively chemotherapy) over 1 to 3 years. High risk patients included Ta grade 3 tumours, T1 grade 2 or 3 tumours, primary carcinoma in situ (CIS) without evidence of urothelial carcinoma, or concurrent CIS in several localisations. Patients who had 3 or more separate tumours diagnosed within 18 months of follow-up or recurrences at multiple sites at first or second follow-up following TUR also received instillation treatment.

### Grading of urothelial carcinomas

All specimens were independently reviewed according to the WHO73 classification (grades 1 through 3) and WHO04 (low grade or high grade) by experienced pathologists (EG, RW, OM). Two of the pathologists (EG and OM) repeated the classification at a later stage. The pathologists did their evaluations in separate sessions, independently and without prior knowledge of the original stage, grade, each other's assessments, their own assessments, treatment or follow-up of the patients. In case of discrepancies, consensus was reached after discussion using a multihead microscope.

The WHO04 low grade tumours were also reviewed with regards to discerning low grade and PUNLMP tumours.

### Patient follow-up

Follow-up data were retrieved from medical records and from any available new specimens at the Department of Pathology, SUH.

The follow up protocol depended on the grade and stage of the primary tumour. Provided that follow up cystoscopies were negative, patients with Ta grade 1 tumours would undergo cystoscopy 3 months after initial diagnosis followed by cystoscopy after 9 months and consequently annually for 5 years. All other patients would have cystoscopies every 3 months for the first 2 years, every 4 months the 3^rd^ year, every 6 month the 4^th^ and 5^th^ years followed by annual cystoscopies thereafter.

Recurrence was defined as the reappearance of histopathologically confirmed urothelial carcinoma in the bladder. Progression was defined as an advance in stage, histologically proven metastasis or death of disease.

### Statistical analysis

The inter- and intra-observer reproducibility was measured by unweighted or quadratically weighted kappa statistics as appropriate. Unweighted kappa statistics were used for dichotomized variables (WHO04 and WHO73 (1&2 vs. 3)). Weighted kappa statistics were applied for WHO73 as this classification system has 3 categories. Quadratic weight, rather than linear weight, was used as the difference between the second and third category (grade 2 and 3) has greater clinical implications than the difference between the first and second category (grades 1 and 2). To evaluate the consistency of the grading systems, mean grade was calculated [12].

For comparison between different groups of patients, log rank test, Kaplan-Meier survival curves and the Mann Whitney U test were used. Median ages for patients with different WHO73 or WHO04 grade tumours were compared by Mann Whitney U tests. Log rank tests were used to compare survival times between the groups of patients. The reported p-values are two-sided, i.e. the null hypothesis is that there is no difference between the groups and the alternate hypothesis that there is a difference. As a measure of predictive discrimination of those who did vs. those who did not experience progression within 5 years, we present sensitivities, specificities, and positive and negative predictive values (PPV and NPV). Continuity corrected confidence intervals were estimated. Positive predictive value was defined as patients with stage progression and high grade or grade 3 tumours (true positives) divided by the total number of true positives and patients with high grade (or grade 3 tumours) who did not experience progression (false positives). Conversely, negative predictive value was defined as patients without stage progression (true negatives) divided by the total number of true negatives and patients with low grade (or grade 1 and 2 tumours) who did experience progression (false negatives). Sensitivity was defined as true positives divided by the total number of patients with progression, and specificity was defined as true negatives divided by the total number of patients without progression.

The time of progression was considered in survival analyses, using Kaplan-Meier plots and univariable Cox proportional hazards models. The proportional hazards assumption was tested by inspection of stratified log minus log survival plots and by introducing time-dependent covariates into the models. The predictive ability with regard to time of progression was measured by Somers' D rank correlation R function rcorr.cens of the package Hmisc, which was transformed into Harrell's (concordance) C-index by the formula C = 0.5 * (|D|+1) [13]. Confidence intervals for the C-indices were bootstrapped percentile intervals, using simple nonparametric bootstrapping with 2000 samples. Finally, in order to correct for the “optimism” in a concordance measure evaluated on the same data that was used to fit the survival model, adjusted (“bootstrapped”) C-indices were estimated (R function validate.cph of the package rms, with B = 150).

Statistical analyses were performed using IBM SPSS for Windows version 21.0 (IBM Corp, Armonk, NY, USA), VassarStats (http://vassarstats.net) and R Project for Statistical Computing (http://www.R-project.org)

## Results

The median age at diagnosis was 74 years (range 39 to 95). One hundred and forty-eight patients were male (76.7%) and 45 (23.3%) female (ratio = 3.3). Median age depended on WHO73-grade and was 65.5 years for grade 1, 74.0 years for grade 2 and 75.0 years for grade 3 tumours (Mann Whitney U tests gave p = 0.005 for grade 1 versus 2, p = 0.22 for 2 versus 3). For WHO04, the median age was 71.0 years for low grade and 75.5 years for high grade tumours (p = 0.006).

Median follow-up time was 75 months (range 1–127). Histologically proven recurrences occurred in 111 patients (57.5%). Stage progression at recurrence occurred in 17 patients (15.3% of the patients with recurrence or 8.8% of all patients), 14 of these within 36 months and 3 more than 5 years (at 61, 62 and 101 months) after the original diagnosis. We used recurrence and progression within 5 years after the initial diagnosis as the endpoint because of the obvious dichotomous progression pattern (<3years versus >5 years), and also as it seemed unlikely to us that biomarkers can predict progression after such a long interval.

The excluded patients had a median age of 75 years (range 49–90). 71% were male, 29% female. Median follow-up time was 35 months (0–137 months). There were no differences in sex, age, stage, initial diagnosis (grade), or occurrence of carcinoma in situ between the excluded and the included patients. Of the excluded patients, 36 had true non-muscle-invasive urothelial carcinomas with adequate follow-up. Of these, 28% recurred and 14% progressed to a higher T-stage.

### Reproducibility

The distribution of consensus WHO73-grades 1, 2 and 3 was 44 (23%), 98 (51%) and 51 (26%). One hundred and nineteen tumours (62%) were low grade and 74 (38%) were high grade according to the WHO04. For the final consensus WHO73 grades, there was pre-discussion consensus between the reviewing pathologists on the grade of 39 of the grade 1 tumours (88.6%), 55 of the grade 2 tumours (56.1%), and on 34 of the grade 3 tumours (65.4%). Regarding the final consensus WHO04 grades, there was pre-discussion agreement on 119 of the low grade (100%) and 49 of the high grade tumours (66%). On consensus diagnosis, all WHO73 grade 1 tumours were classified as low grade. Twenty-four (24.5%) of the grade 2 and 50 (98.0%) of the grade 3 tumours were re-classified as high grade tumours, and one grade 3 downgraded to a low-grade tumour.

Tables 2 and 3 summarize inter- and intraobserver overall agreement and kappa-values with 95% confidence intervals (95% CI). The interobserver reproducibility of the WHO73, both as three-tiered and grades 1&2 versus 3, and the WHO04 were very similar with overlapping confidence intervals. For pathologist 1, intraobserver reproducibility for the WHO73 (both two-tiered and three-tiered) is very similar to the interobserver reproducibility. For pathologist 2 there is more variation as the WHO73 (three tiered) seems less reproducible and the WHO04 more reproducible for this observer than the interobserver reproducibility, however wide and overlapping confidence intervals makes a clear-cut conclusion difficult.

**Table 2:** Interobserver reproducibility.

	Overall agreement (95% CI)	Kappa (95% CI)
**WHO73**	66% (59–73%)	0.68 (0.57–0.78)*
**WHO73 (1&2 vs. 3)**	89% (83–93%)	0.68 (0.56–0.80)
**WHO04**	87% (81–91%)	0.70 (0.59–0.81)

: Quadratic weighted kappa.

CI: Confidence interval.

**Table 3:** Intraobserver reproducibility.

	Pathologist 1		Pathologist 2	
	Overall agreement (95% CI)	Estimated kappa (95% CI)	Overall agreement (95% CI)	Estimated kappa (95% CI)
**WHO73**	68% (61–74%)	0.69 (0.59–0.79)*	63% (56–70%)	0.61 (0.48–0.74)*
**WHO73 (1&2 vs. 3)**	88% (82–92%)	0.66 (0.54–0.79)	89% (83–93%)	0.68 (0.55–0.80)
**WHO04**	Not performed	Not performed	93% (88–96%)	0.83 (0.74–0.92)

: Quadratic weighted kappa.

CI: Confidence interval.

In our study, only one pathologist assessed both grading systems (OM). The mean grade difference for this pathologist is 0.3 grade points in both grading systems (table 4). For the other pathologist who did to reviews of a grading system (EG, WHO73), the mean grade difference was 0.4. Due to the very low number of PUNLMPS, direct comparison of the mean grade of the two systems is not feasible.

**Table 4:** The difference in mean grade between the reviewers.

	EG 1	EG 2	RW 1	OM 1	OM 2
**WHO73**	1.83	1.79	NP	2.00	1.97
**WHO04**	NP	NP	2.33	2.31	2.31

EG: Pathologist 1, 1^st^ review. EG2: Pathologist 1, 2^nd^ review. RW 1: Pathologist 2 (only one review). OM: Pathologist 3, 1^st^ review. OM2: pathologist 3, 2^nd^ review. NP: Not performed.

### Prognostic comparison

The patients' age was a statistically significant factor for progression, (p = 0.004), with median time to progression 8 months for patients ≤73 years and 24 months for patients >73 years,), but not for recurrence (p = 0.14). Gender was not prognostically significant (recurrence: p = 0.88; stage progression: p = 0.96).

The recurrence rates of the three WHO73 grades were 57%, 46% and 61% after 5 years. There were no significant differences in recurrence rates of the WHO73, the WHO73 as grades 1&2 versus 3 or the WHO04 high and low grades (51% and 54%, p = 0.25), table 5. The progression-free survival rates for grades 1, 2 and 3 were 95%, 97% and 82% at five years after the index specimen. The progression rate of grade 3 cases differed (p = 0.001) from grades 1 or 2, but the progression rates between grades 1 and 2 did not (p = 0.70).

**Table 5:** Recurrence free survival at 5 years.

	Threshold	Recurrence/patients n (%)
**WHO73**	Grade 1	25/44 (57)
	Grade 2	45/98 (46)
	Grade 3	31/51 (61)
**WHO73 (1&2 vs. 3)**	Grades 1&2	70/142 (49)
	Grade 3	31/51 (61)
**WHO04**	Low grade	61/119 (51)
	High grade	40/74 (54)

CI: Confidence interval.

Stage progression of the WHO04 low and high grades differed greatly (3% versus 15%, p = 0.003). Sensitivity, specificity, hazard ratio (HR), p-values and Harrell's C-index for stage progression-or-not of WHO73 and WHO04 are summarized in Tables 6–7. With very similar Harrell's C-indices, none of the grading systems is prognostically stronger than the others with regard to time to stage progression. The PPV and NPV for the two classification systems are similar with overlapping confidence intervals upholding that none of the grading systems is stronger than the other for predicting stage progression, The specificity of the WHO73 (1&2 vs. 3) is somewhat better than the WHO04. The sensitivity of the WHO04 seems better than for the WHO73 (1&2 vs. 3), but 95% confidence intervals are wide and overlapping making the conclusion ambiguous.

**Table 6:** Progression free survival at 5 years.

	Threshold	Progression/patients, n (%)	HR (95% CI)	Wald p	Harrell's C-index (95% CI)	Harrell's C-index Boot-strapped
**WHO73**	Grade 1	2/44 (5)	0.71 (0.12–4.23	0.010	0.70 (0.53–0.84)	0.68
	Grade 2	3/98 (3)	4.34 (0.94–20.1)			
	Grade 3	9/51 (18)				
**WHO73** (1&2 vs. 3)	Grades 1&2	5/142 (4)	5.42 (1.82–16.2)	0.003	0.70 (0.56–0.83)	0.69
	Grade 3	9/51 (18)				
**WHO04**	Low grade	3/119 (3)	6.59 (1.84–23.6)	0.004	0.72 (0.60–0.82)	0.71
	High grade	11/74 (15)				

CI: Confidence interval. HR: Hazard ratio.

**Table 7:** Sensitivities, specificities, positive and negative predictive values of 5 years progression of the WHO73 (1&2 vs. 3) and WHO04.

	Sensitivity (95% CI)	Specificity (95% CI)	PPV (95% CI)	NPV (95% CI)
**WHO73** (1&2 vs. 3)	64% (36–86%)	77% (70–82%)	18% (9–31%)	96% (92–99%)
**WHO04**	79% (49–94%)	65% (57–72%)	15% (8–25%)	97% (92–99%)

CI: Confidence interval. PPV: positive predictive value. NPV: Negative predictive value.

**Figure 2:**
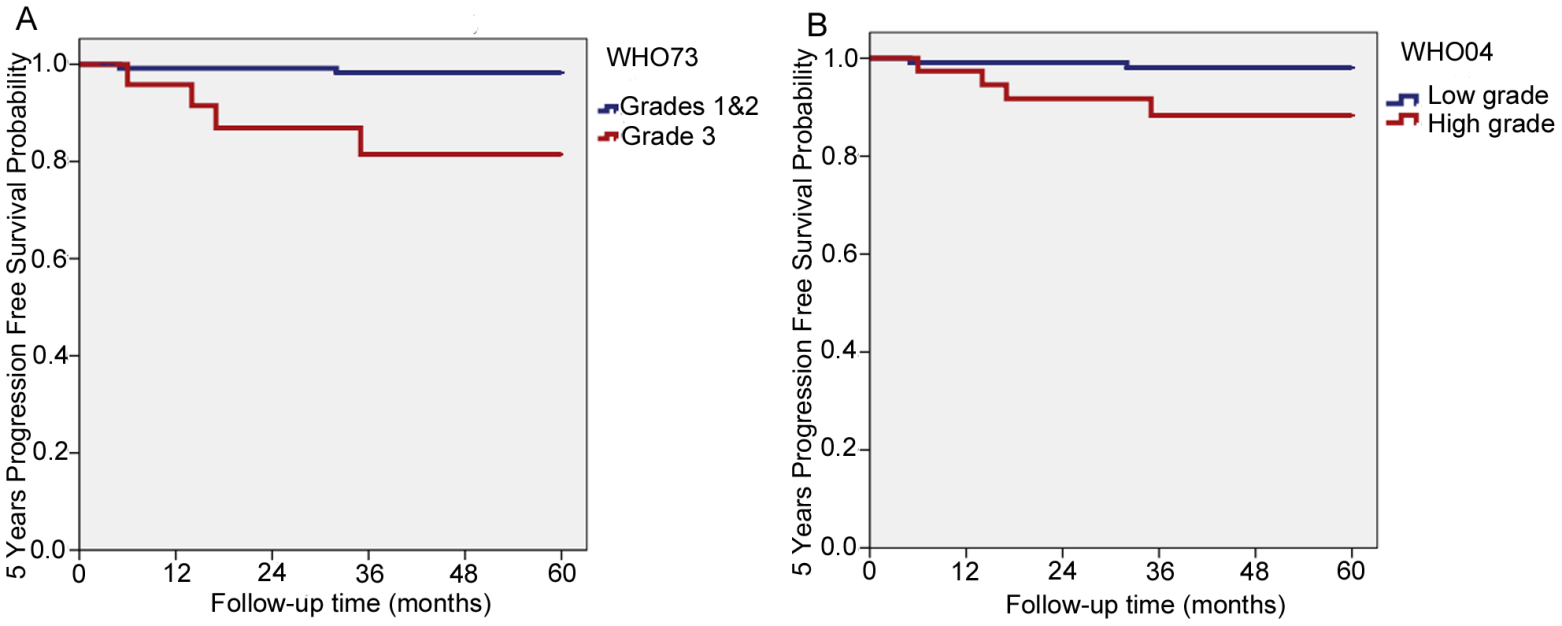
Five years progression free survival, pTa. **A.** WHO73 Grades 1&2 versus 3. **B.** WHO04 Low grade versus high grade.

There were 154 pTa (80%) and 39 pT1 (20%) tumours, with 79 (51%) and 22 (56%) recurrences (p = 0.22) and 6 and 8 progression cases respectively (4% and 20%, p<0.001, HR = 7.0, 95% CI = 2.4–20.3). When analysing progression in the two stages separately, for pTa tumours there were significant differences between the grades of both the WHO73 (grades 1&2 versus 3) and the WHO04, but more so in the WHO73 (p<0.001 versus p = 0.015, Figure 2). There were no significant differences between the progression rates of the different grades in pT1 tumours.

The progression free survival rates (PFSR) of WHO73 grades 1&2 (n = 142) and WHO04 low grade (n = 117) overlapped (PSFR = 97% and 96% respectively), although the number of grades 1&2 was much higher than the WHO04 low grades. As expected, the WHO73 grades 3 had a worse PFSR (82%) than the WHO04 high grades (85%).

### Papillary urothelial neoplasms of low malignant potential

Three cases were classified by the reviewing pathologists as PUNLMP, two by the first and another case by the other. When the cases were evaluated independently by two other pathologists, one of these three cases was classified as low grade, leaving 2 cases as undeniable PUNLMP. The recurrence rate was 50% and none showed stage progression. Comparison with the original WHO grades showed that the 2 PUNLMPs had been classified by all pathologists as WHO73 grade 1 or WHO04 low grade at review. The 44 grade 1 cases recurred in 57% and 4% showed stage progression, which was not statistically different from the 2 PUNLMPs (p = 0.64 and p = 0.75). Of the low grade tumours, 58% recurred and 3% showed stage progression. Recurrence and stage progression in the PUNLMPs and the low grade tumours by univariate survival analysis were not different (p = 0.81 and 0.79).

## Discussion

The clinical course of urinary bladder cancer is strongly heterogeneous. Tumour stage is the most important classical clinicopathological parameter for the prognosis of urothelial carcinoma of the urinary bladder, but the extent of invasion is hard to determine on (superficial) biopsies alone. Additional prognostic value is obtained by the histology of the tumour. Well differentiated urothelial carcinoma usually grows superficially, while poorly differentiated urothelial carcinoma more often has an infiltrating growth pattern at the time of presentation. In 2006, the European Organization for Research and Treatment of Cancer (EORTC) developed a scoring system for risk of recurrence and progression [14]. The system is based on the following factors: tumour size, number of tumours, prior recurrence rate, histological grade and stage, and the presence of concomitant carcinoma in situ. These variables are weighted and the combined score determines risk stratification of the patients. High risk patients are routinely treated with BCG-instillations. The EORTC risk scores have not been calculated in our study. The clinical information was extracted from patient records several years after the treatment was given. For a large proportion of the patients the tumour size or number of tumours was not recorded, hence the risk score is impossible to calculate.

The WHO73 grading system is a well-established and accepted system. There have however been discussions over the reproducibility of this system, and the WHO04 grading system was designed and hoped to be universally acceptable and better reproducible. However, several studies have shown considerable inter-observer variability using the WHO04 as well. In our material, the WHO73 (as grades 1&2 versus 3) and WHO04 (as low grade versus high grade) have nearly the same interobserver reproducibility, which was not perfect, and slightly more variation in the intraobserver reproducibility for one of the observers as the WHO04 is possibly better reproducible than the WHO73. The WHO04 has many more high-grade tumours (n = 74) than the WHO73 (grade 3, n = 51). None of the grading systems is superior with regards to predicting recurrence or stage progression.

The current European guidelines on treatment of non-muscle-invasive bladder carcinoma recommends reporting according to both grading systems as the clinical guidelines are based on the WHO73 but the WHO04 is also used. The improvement of the 2004 classification has been disputed by several authors with the main debate being the PUNLMP [8], [15]–[18]. A two-tier system differentiating only between low grade and high grade tumours would yield better reproducibility results. The WHO04 has not been implemented in the clinical guidelines as the predictive value of the grading system with respect to recurrence and stage progression is not yet fully investigated.

Table 8 summarizes the results from the largest study on WHO04, by Pan et al [15], a recent study comparing the WHO73 and the WHO04 grading systems by Chen et al [19] and our study. In Pan's study a very large number of cases were evaluated, but it does not compare the WHO73 with the WHO04. Moreover, the WHO04 evaluations have been done by one pathologist only. Chen et al (2012) did compare both grading systems, but again only one pathologist did the review. We used three independent reviewers. Statistically, none of the grading systems was stronger than the others with regards to predicting recurrence or progression in our study. The proportion of WHO73 grade 3 tumours was 26% whereas there were 38% WHO04 high grade tumours, both considerably higher than the overall progression rate of 9%. For patients with otherwise similar risk factors for progression (multiplicity, tumour size, progression rate, and stage), this could lead to overtreatment if the WHO04 high grade tumours were treated similarly to the WHO73 grade 3 tumours. This could point to a slight preference for the WHO73. In addition, Table 6 shows that there are considerable differences between the populations from different countries. Multicentre international studies are needed to give a better impression about the real value of the WHO73 and WHO04; however, a more reasonable conclusion would be that both systems have a very low predictive value. It therefore seems better to study new molecular quantitative biomarkers which may have stronger prognostic value and also can be better reproducible than conventional microscopic evaluations, as has been found for Ki67 in breast cancer [20]. In 2010, van Rhijn et al showed that using a molecular grade, consisting of a combination of FGFR3 mutations status and Ki67%, could predict recurrence and progression more accurately and more reproducibly [21]. Combining the molecular grade and the EORTC risk score could provide clinicians with an even more precise tool for therapy decision making with regard choice of follow-up and treatment.

**Table 8:** Comparison of the studies by Pan et al (2010), Chen et al (2012) and Mangrud et al (2013).

	Pan	Chen	Mangrud
**Period**	1991–2005	1999–2009	2002–2006
**Time**	15 years	10 years	5 years
**Patients**	2191	392	249
**Included**	1515	348	193
**Men**	1307 (86%)	287 (82.5%)	148 (76.7%)
**Women (%)**	208 (14%)	61 (17.5%)	45 (23.3%)
**Mean age**	71 (23–92)	N/A	71 (39–95)
**Median age**	N/A	68 (21–92)	74 (39–95)
**Reviewers**	1	1	3
**Grading system**	WHO04	WHO73/WHO04	WHO73/WHO04
**Patients with complete follow-up**	874	?	193
**Median follow-up, months**	74 (1–215)	47 (2–124)	75 (1–127)
**IVI Treatment** *	592 (39%)	?	35 (18%)
**PUNLMP**	212 (14.0%)	40 (11.5%)	2 (1.0%)
**Low grade**	706 (46.6%)	223 (64.1%)	117 (61%)
**High grade**	597 (39.4%)	85 (24.4%)	74 (38%)
**Grade 1**	N/A	125 (35.9%)	44 (23%)
**Grade 2**	N/A	176 (50.6%)	98 (51%)
**Grade 3**	N/A	47 (13.5%)	51 (26%)
**pTa**	1006 (66.4%)	220 (63.2%)	154 (80%)
**pT1**	509 (33.6%)	128 (46.8%)	39 (20%)
**Recurrence, total**	484 (31.9%)	122 (35.1%)	111 (57.5%)
**Recurrence, PUNLMP**	17.9%	25.0%	50%
**Recurrence, low grade**	35.0%	30.0%	59%
**Recurrence, high grade**	34.0%	52.9%	56%
**Recurrence, grade 1**	N/A	15.2%	57%
**Recurrence, grade 2**	N/A	42.0%	46%
**Recurrence, grade 3**	N/A	61.7%	61%
**Progression, total**	222 (14.7%)	41 (11.8%)	17 (8.8%)
**Progression, PUNLMP**	1.9%	0.0%	0%
**Progression, low grade**	6.5%	6.7%	3%
**Progression, high grade**	28.8%	30.6%	15%
**Progression, grade 1**	N/A	2.4%	4.5%
**Progression, grade 2**	N/A	27.0%	3.1%
**Progression, grade 3**	N/A	38.3%	18%
**Progression, definition**	Advanced stage, metastasis or death.	pT2 or higher	Advanced stage, metastasis or death.

IVI Treatment: Intra-vesical instillation treatment.

N/A: Not applicable.

The median age in our study population was somewhat higher than reported by others [6], [22], but in Norway, the median age at diagnosis of new cancers (primary diagnosis) of the bladder, urethra and ureters in Norway in 2005–2009 falls within the 70–74 years bracket [23]. Although the median age might be higher than in other publications, the population of the catchment area of SUH does not differ from the Norwegian population as a whole.

The clinical impact of PUNLMP is not yet established. As reproducibility is low, recurrence and progression rates should be interpreted with caution. Recurrence rates vary from 3 to 60% [24], including several patients who have been diagnosed with muscle-invasive carcinoma at a later stage [25]. The follow-up of this group is controversial. Some authors believe they should be grouped with the low grade carcinomas, as recurrence rates and disease-specific mortality rates do not differ significantly [18], [26]. However, there are also authors who argue that patients with PUNLMP need not be followed as closely as patients with low grade urothelial carcinomas as some studies show low recurrence rates for PUNLMP [17]. Others add to this by arguing that the psychological trauma may be less if the patients are not given a cancer-label. This view has not been validated in clinical trials. Avoiding the cancer-label might be of importance in areas where universal health care is not available, and a history of cancer may be negative with regards to insurance issues [16].

PUNLMPs are very rare in our material. Other studies report PUNLMP-rates of 12–39%. As specimens from all the patients treated for bladder cancer in the South Rogaland region are sent to our laboratory, ours is a population based material. Both urothelial carcinomas and papillomas from the study period were reviewed to ensure that no PUNLMPs were falsely labelled as papillomas. The very low incidence therefore seems real and representative for our region. One could hypothesise that the scarcity of PUNLMPs could be due to the tendency of a rather medically conservative attitude in the population of the catchment area of our hospital as preventative screening for bladder carcinoma is not performed, and investigation and diagnosis depend on clinical presentation.

In conclusion, there are still challenges with respect to reproducibility and specificity to predict stage progression. We propose further studies of the additional value of quantitative molecular biomarkers such as proliferation markers (Ki67, PPH3 and FGFR3) and possibly also host immune response to improve the reproducibility and prognostic value of predicting stage progression.
